# Keystone Veterinary Medical Association

**Published:** 1893-12

**Authors:** W. L. Hart

**Affiliations:** Secretary; Philadelphia


### KEYSTONE VETERINARY MEDICAL ASSOCIATION.

The regular meeting of the Keystone Veterinary Medical Associaton was held Nov. 16, 1893. The meeting was claled to order by the President, Dr. R. A. Webster.

There answered roll call: Drs. Hoskins, Webster, Bridge, Lintz, Gœntner, Kooker, Weber, J. R. Hart, W. L. Hart.

The minutes of the previous meeting were read and approved.

The application of Dr. W. L. Rhodes, Lansdowne Pa., (American Veterinary College,) was received for membership, and was referred to Trustees to be reported at next meeting.

It was moved by Dr. Hoskins and seconded, that the Board of Censors be authorized to look after the Prothonotary office, to make the necessary changes and notify all the veterinarians to register.

Dr. Hoskins gave a very interesting report of the Veterinary Congress held in Chicago, for which the Association gave a vote of thanks.

The following were elected officers to serve for the ensuing year: President, Dr. Chas. B. Gœntner; Vice-President, Dr. S. E. Weber; Secretary, Dr. Walter L. Hart; Treasurer, Dr. Francis Bridge.

The next meeting will be held at the office of Dr. Hoskins, 3452 Ludlow Street, second Tuesday in December. Dr. S. E. Weber, essayist.

Philadelphia. W. L. HART, Secretary

